# Fracture resistance of translucent zirconia overlay restoration with different preparation designs

**DOI:** 10.1186/s12903-025-07456-3

**Published:** 2026-01-12

**Authors:** Yousra Ahmed Awad, Lamia Dawood, Mohamed El-Anwar, Ahmed Attia

**Affiliations:** 1 Department of Fixed Prosthodontics, Faculty of Dentistry, Mansoura University, Mansoura, Egypt; 2 Department of Mechanical Engineering, National Research Centre, Cairo, Egypt

**Keywords:** Overlay, Translucent zirconia, Fracture, Preparation

## Abstract

**Background:**

This in-vitro study was planned to evaluate the fracture load of translucent zirconia overlay restoration made with different tooth preparation designs.

**Materials and methods:**

Thirty-two sound mandibular molars were divided into four main groups (*n* = 8), based on different overlay preparation designs. Group 1 anatomic cusps with chamfer finish line (ACC), group 2: flat cusp with chamfer finish line (FCC), group 3: anatomic cusp reduction with proximal steps (ACS), group 4: flat cups reduction with proximal steps (FCS). The teeth were restored using translucent monolithic zirconia (IPS e.max ZirCAD, Ivoclar, FL). Cementation was performed using adhesive resin cement. Specimens were stored in water at 37 °C for 45 days then followed by cyclic loading fatigue for 120,000 cycles. Finally, specimens were fractured under compressive load in Newton (N) using a universal testing machine. A stereo microscope and scanning electron microscope (SEM) were used to examine and categorize the failure pattern modes. Two and one-way ANOVAs and Post hoc Tukey tests were used for statistical analyses.

**Results:**

Group ACC demonstrated the highest fracture resistance (4371 ± 440), followed by ACS group (3995 ± 274) and FCC group (3954 ± 698), FCS group showed the lowest fracture load (3483 ± 567).

**Conclusion:**

Both cusp design and margin design affected fracture resistance. Anatomical cusp with chamfer finish line showed the highest fracture resistance.

## Background

Patients are increasingly concerned with preserving their natural teeth [1]. However, natural tooth substrates loss caused by decay or harmful habits tends to rise with age, making it an active era of research to extend the lifespan of restored teeth [1]. Proper tooth preparation and selection of restorative material are important factors to achieve longevity of restored teeth clinically [2]. Minimally invasive technique is a key principle in modern restorative dentistry to preserve remaining natural tooth substrates while reducing the risk of fracture [3, 4]. Indirect partial coverage restorations can be divided into three groups according to intra coronal cavity and cuspal coverage as follow; inlays (intra coronal cavity without covering any cusps), onlays (intra coronal cavity with covering one or more cusps), and overlays (intra coronal cavity with covering all cusps) [5]. Overlay is an indirect restoration for posterior teeth with mesial-occlusal distal (MOD) cavity portion extended to cover all occlusal surfaces [6].

Posterior indirect resin bonded restorations (PIAR) represent an indirect approach aimed to preserve remaining tooth substrates and enhancing restoration strength [7]. The success of PIAR depends on a durable adhesive bond at restoration/cement/tooth interfaces, to achieve biomimitc recovery of the tooth to withstanding different clinical conditions [8].

Zirconia is an attractive restorative material due to its biocompatibility and excellent strength, however its limited translucency is a common drawback [9]. Therefore, dental manufacturers tried to satisfy the interest for higher esthetic monolithic zirconia ceramics by creating new materials such as translucent, high translucent and super high translucent zirconia [9]. The first generation of dental zirconia, known as 3Y-TZP, used 3 mol % yttria to partially stabilize the tetragonal phase with 0.25 weight% alumina [10]. This composition had the highest fracture toughness (3.5 to 4.5 MPa·m1/2) and a flexural strength of 1200–1500 MPa but its opacity limited its use to posterior applications [11]. An improved, translucent 3Y-TZP zirconia reduced the alumina content from 0.25 weight% to 0.05 weight %, thus maintained a high zirconia’s mechanical properties with increasing its translucency [11].

Fracture resistance is a common test in dentistry used to evaluate fracture resistance of restored teeth in-vitro [12, 13]. Both material properties and preparation design influence ultimate mechanical integrity of restored teeth with resin bonded all-ceramic restorations [14]. Therefore, it is difficult to determine exactly how different preparation designs could affect fracture resistance [14, 15].

Another critical aspect is the cuspal coverage, which significantly affect fracture resistance of resin bonded all-ceramic restorations [16, 17]. Furthermore, marginal fit is a key factor for long-term success of resin bonded all-ceramic restorations [18]. A well-defined margins contribute to a strong and stable interface which is crucial for the restoration’s durability [19]. Before conducting costly clinical studies, in vitro studies should be conducted to predict the viability of new materials and techniques to be used clinically [20, 21]. However, in-vitro studies should mimic clinical conditions, therefore storage in water and cyclic loading fatigue are widely used to simulate clinical conditions [22, 23].

The hypothesis of this in-vitro study was that both cusp design and margin design would affect fracture resistance of molars restored with monolithic translucent zirconia overlay restorations.

## Materials and methods

Sample size calculation was based on mean fracture resistance of CAD/CAM crowns retrieved from previous studies [20, 21]. Using G power program version 3.1.9.7 to calculate sample size based on effect size of 2.58, using 2-tailed test, α error = 0.05 and power = 95.0%, the total calculated sample size will be 6 in each group and by adding 20% to compensate for possible drop out then total sample size per group will be 8 in each test group.

All procedures performed in this study, were carried out in accordance with relevant guidelines and regulations of Helsinki Declarations. All the experimental protocols were approved by the ethical committee at Faculty of Dentistry, Mansoura University with reference number (M0104023 FP). A total of thirty-two sound human mandibular molars extracted due to periodontal problems were collected from patients who needed full dentures or had diabetes. Teeth have been obtained after the patient’s agreement from the Faculty of Dentistry at Mansoura University’s Oral and Maxillofacial Surgery Department. All teeth were meticulously examined using 5X magnification magnifying loupes (Univet, Italy). A caliber was used to measure both BL and MD dimensions of each molar. Molars that considerably exceeded the average dimensions of 9 ± 1 mm buccolingual and 10 ± 1 mm mesiodistal width were excluded. After removing calculus and soft tissues remnants with an ultrasonic scaler (UDS-K, Guilin Woodpecker Medical Instrument Co. Ltd., Guabgxi, China). To prevent dehydration teeth were sterilized for a week with 5.25% sodium hypochlorite diluted 1:10 at room temperature and maintained in distilled water throughout the study. The water was routinely replaced every 5 days. First the root portion, 2 mm below cemento-enamel junction was coated with an artificial periodontal membrane made from a gum resin (AntiRutsch-Lack; Wenko-Wenselaar, Hilden, Germany) [20, 24].

### Molars preparation

All teeth were divided into four test groups (*n* = 8) and received MOD cavity preparation according to principles of tooth preparation for ceramic inlay with pulpal depth and width 2 mm for all group [25]. The amount of cusp reduction was measured using a silicon index that had already been fabricated (Silaxil Condensation Silicon, Rubber Base, Lascode, Italy). Axial preparation was done using a dental surveyor (Surveyor Type II saeshin, korea) and occlusal reduction was performed using high speed handpice (PANA-AIR, Japan) under air-water coolant system.

**Fig. 1:**
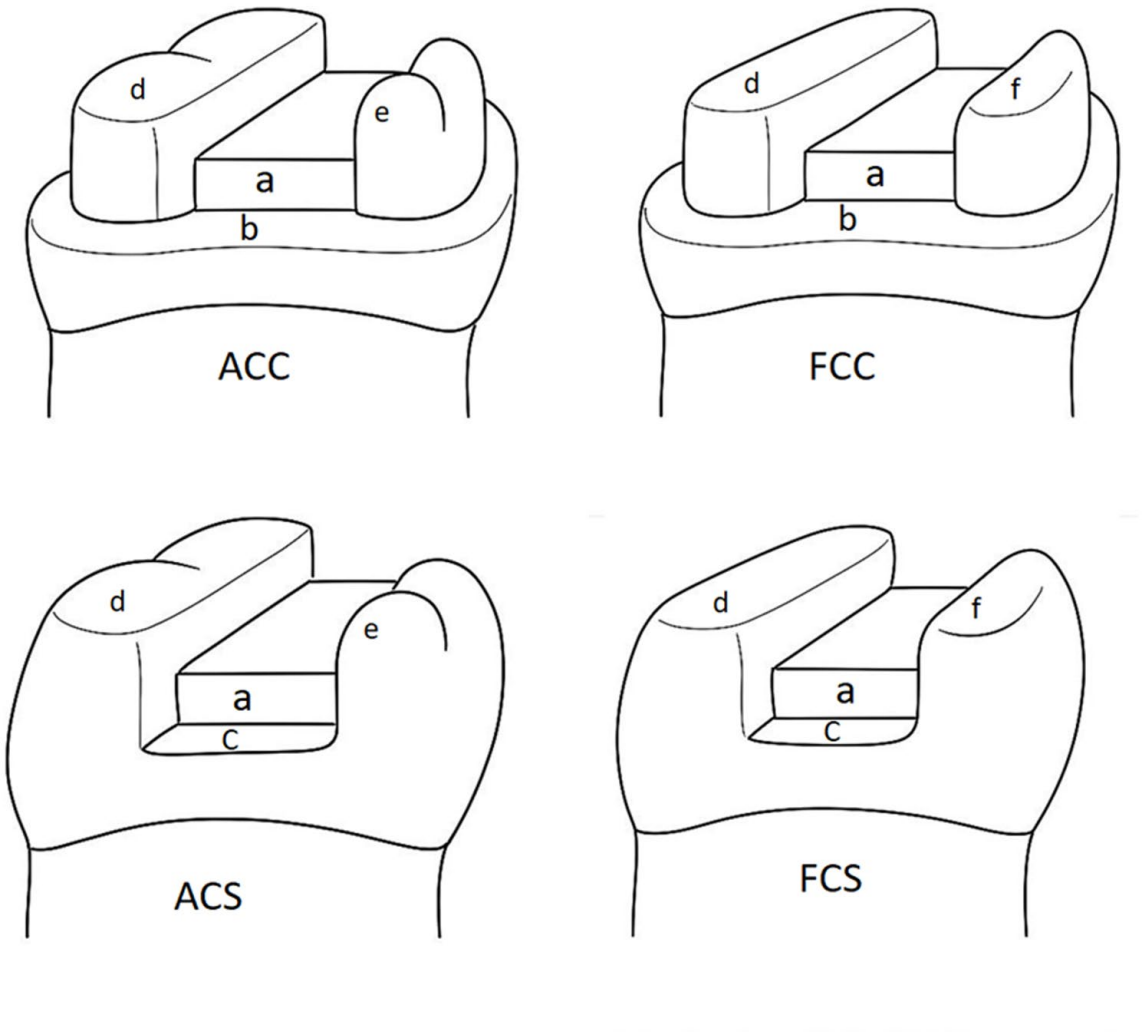
Schematic diagram of different preparation designs A. 1 m. B. 0.5 mm. C. 1 mm. D. 1.5 mm. E. 1 mm & F. 1.5 mm

Preparation of teeth for overlay restorations, cusps were reduced using two main designs anatomical cusp reduction and flat cusp reduction [26]. All sharp internal angles were rounded and smoothed using finishing stone (Komet Medical, Lemego, Germany) to minimize stress distribution on the bonded restoration and at restoration/tooth interface. Details of the four preparation designs are illustrated in (Fig. 1) and (Table 1).

**Table 1:** Details of Preparation design of all test groups

Test group (*n* = 8)	Function cusp Reduction	Non-function cusp Reduction	Axial wall	Margin design
Anatomical cusp reduction with chamfer finish line (ACC)	1.5 mm	1 mm	6°	0.5 mm chamfer finish line
Flat cusp reduction with chamfer finish line (FCC)	1.5 mm	1.5 mm	6°	0.5 mm chamfer finish line
Anatomical cusp with mesial and distal steps (ACS)	1.5 mm	1 mm	6°	Mesial and distal steps 1 mm depth below pulpal floor with width 1 mm.
Flat cusp with mesial and distal steps (FCS)	1.5 mm	1.5 mm	6°	Mesial and distal steps 1 mm depth below pulpal floor with width 1 mm

### Overlay restoration fabrication

For optical and accurate scans, prepared teeth were scanned using the intraoral scanner (MEDIT i500, Medit Company, Seoul, South Korea). Software design (Exocad, Rijeka 3.1 CAD, GmbH, Darmstadt, Germany) was used for designing overlay restoration with standard restoration’s thickness between 1 mm and 1.5 mm, 0.5 at finish line, according to the study designs. Based on the selected preparation thickness and modified to be equal from cusp and fissure. The cement gap was established at 0.5 mm above cervical margins [27]. A 5 axis milling machine (CORITEC I50i PRO, Eiterfeld Hessen, Germany) was used for dry-milling of overlay restorations from monolithic translucent zirconia (3Y-TZP), (IPS e.max ZirCAD, Ivoclar-Vivadent, FL). Chemical composition of zirconia blanks as provided by the manufacturer (Zirconium Dioxide (ZrO_2_): 87% – 95%, Yttrium Oxide (Y_2_O_3_): 4% − 6%, Hafnium Dioxide (HfO2): 1% – 5%) (Table 2).

**Table 2:** Materials used in the present study

Material Type	Product name	Main Chemical Composition	Manufacture	LOT number
IPS e.max ZirCAD	Monolithic translucent zirconia 3Y-TZP	ZrO_2_ + HfO_2_ + Y_2_O_3_ 95% mass percentage% Y_2_O_3_ (Yttrium oxide) 4–6 HfO_2_ (Hafnium oxide) 5% Al_2_O_3_ Aluminium oxide 1%	Ivoclar Vivadent AG, Schaan, Liechtenstein	Z05Z5C
Monobond N	Universal primer	Alcohol solution of silane methacrylate, phosphoric acid methacrylate, and sulphide methacrylate	Ivoclar Vivadent AG	Z04S5G
Multilink N Primer A and B	Dental adhesive	Primer A: is an aqueous solution of initiators. Primer B: contains HEMA, phosphonic acid and methacrylate monomers.	Ivoclar Vivadent AG	Z056YL
Multilink N	Dual-cure adhesive resin cement in syringe	The monomer matrix is composed of dimethacrylate and HEMA. The inorganic filler includes barium glass, ytterbium trifluoride, and spheroid mixed oxide.	Ivoclar Vivadent AG	Z056YL

Restorations were glazed using furnace (Program at EP 3010) in accordance with the manufacturer’s guidelines. Intaglio surfaces of overlays were air borne particle abraded with 50 μm alumina particles applied for 10 s under pressure of 2 bar at distance 10 mm between the sandblast tips and overlay restoration [28]. Finally, overlays were ultrasonically cleaned in 99% alcohol for 3 min and dried carefully by air [21, 22].

### Bonding of overlays

All bonding steps were carried out according to the manufacturer instructions of the universal primer and adhesive resin cement used for definitive bonding as follow; Universal primer (Monobond N), was applied to the intaglio surface of each overlay restoration using a micro-brush and was rubbed for 60 s, then dried gently for 5 s. All prepared teeth were treated with a self-etching primer Multilink Primer A and B, which was mixed in a ratio of 1:1 for 10 s using a microbrush. The mix was then applied to the bonding tooth surface for 30 s. The conditioned teeth surfaces were dried with oil and water free air spray, leaving the surfaces appearing glossy [29].

### Load application

Each specimen was secured to a custom-made static load device, which was designed to apply a consistent and uniform compressive load. To simulate occlusal forces during the bonding, a vertical load of 5 kg was applied to the bonded overlay/tooth assembly. Metal ball with 5 mm in diameter was centralized on the occlusal surface contacting both buccal and lingual cusps to apply the vertical load. This load was maintained for a duration of 5 min to ensure complete setting of the resin cement [30]. Excess luting cement was removed using a micro brush, and all specimens were light polymerized for 40 s. on each tooth’s surface with an LED light curing system (Coltolux LED, Coltene/Whaledent Inc., Switzerland) with an average light intensity of 1000 mW/cm2.

### Artificial aging

After one hour all bonded specimens were stored in water at 37˚C for 45 days in water proof plastic pots. Finally, all specimens were fatigued in a computerized masticatory simulator (Willitec Kausimulator Version 3.1.3; Willitec, Munich, Germany) under wet conditions for 120,000 cycles to mimic one year clinically [31]. The loading cycle frequency was 1.2 Hz, with a kinetic energy of 2,250 × 10–6 J, maximum load of 49 N, and a minimum load of 0 N. Steatite ceramic balls (4 mm diameter; Hoechst Ceram Tec, Wunsiedel, Germany) were used as antagonistic surfaces to simulate the opposite teeth [20, 21]. All specimens were visually evaluated after cyclic loading to check for cracks.

### Fracture load testing

Specimens were loaded using a universal testing device (Instron 3345, USA) (Bluehill Universal Software, Instron, USA) until the first failure either crack or fracture. A stainless steel bar with a 4-mm diameter ball end was positioned in the middle of the central fossa and contacting buccal and lingual cusps with a crosshead speed of 0.5 mm/min. The fracture load of each specimen was recorded in Newton (N) [20].

### Failure pattern evaluation

**Fig. 2:**
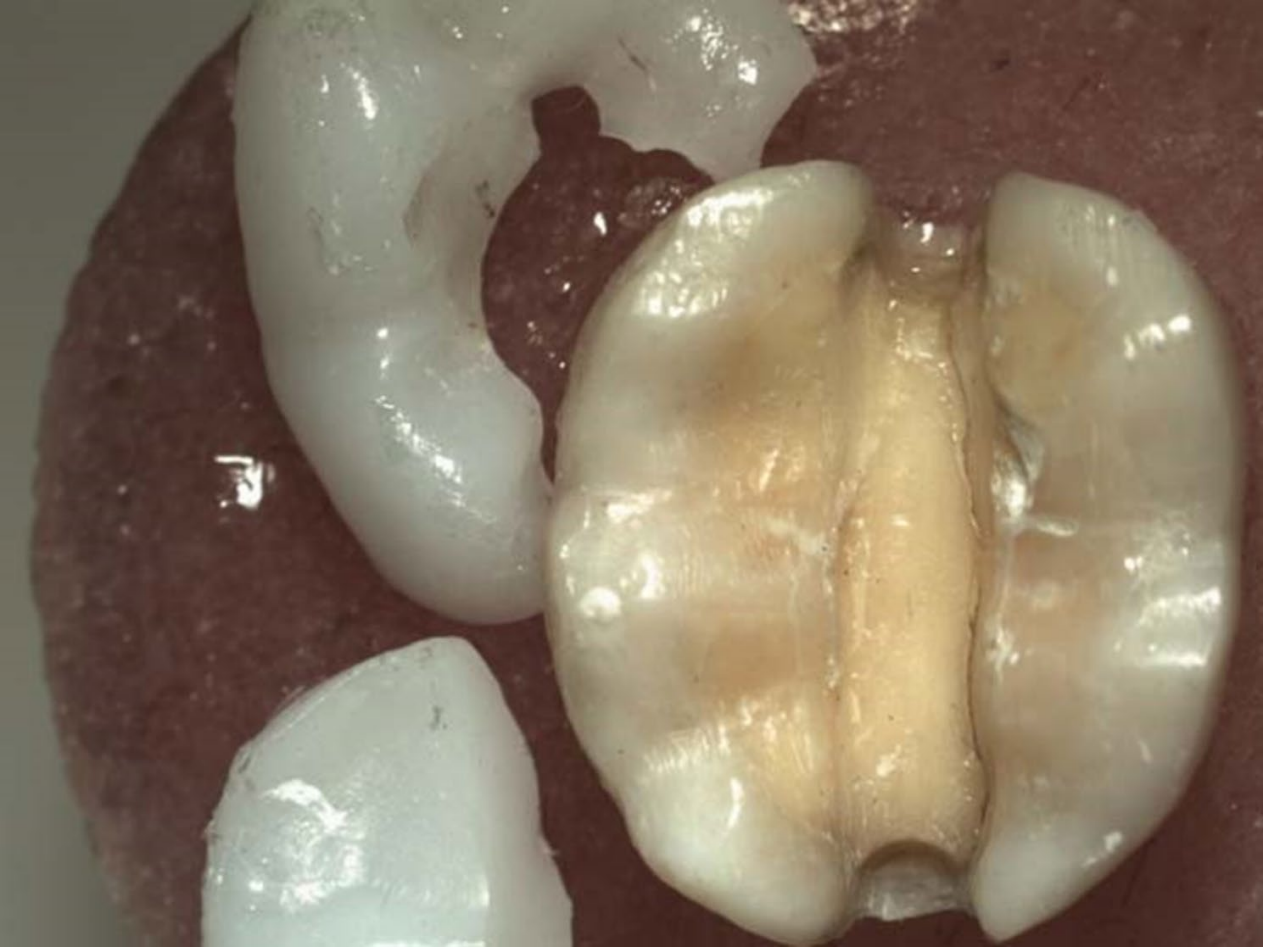
Type I failure pattern; Fracture of the restoration only

**Fig. 3:**
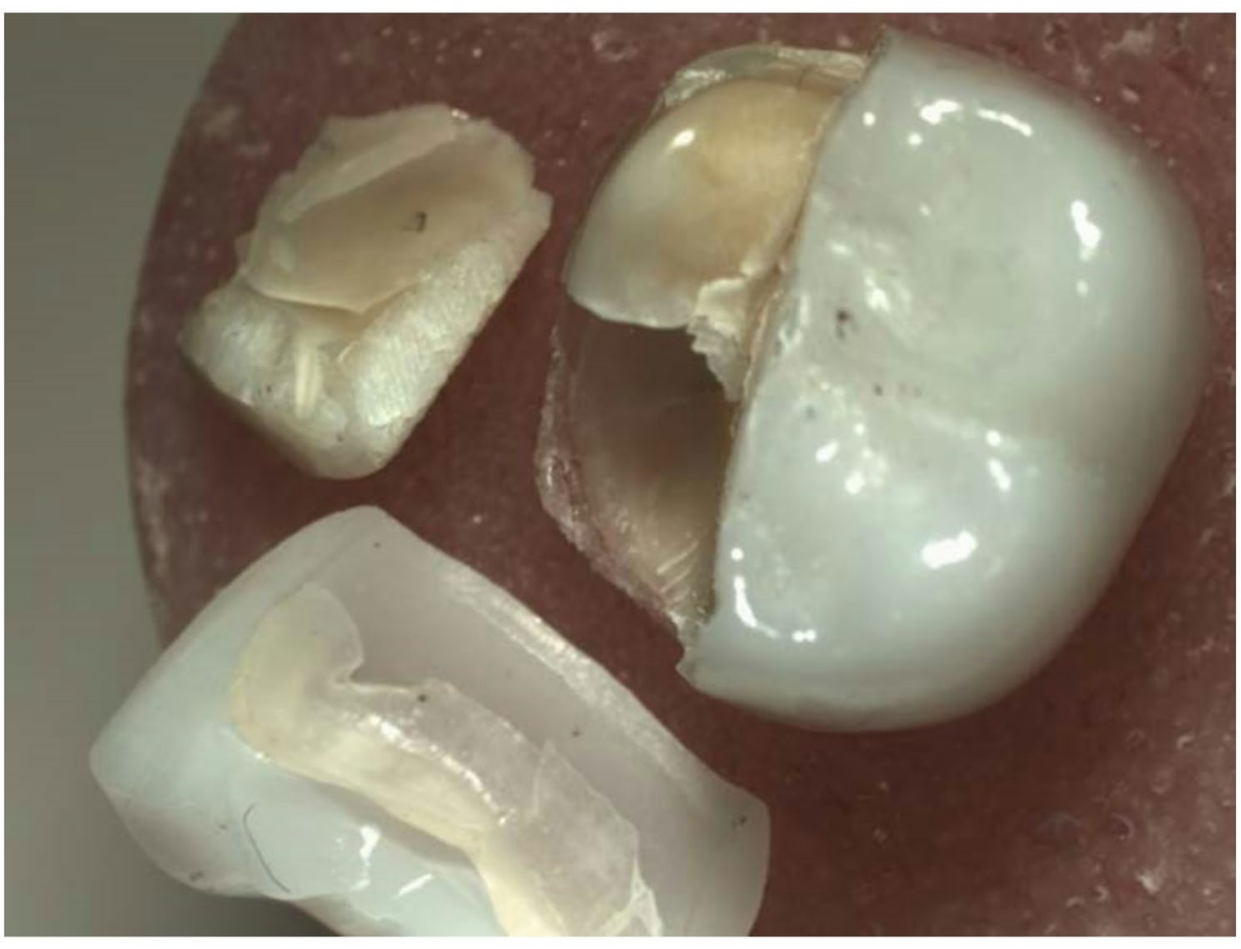
Type II failure pattern; Fracture of the restoration and underlying tooth

**Fig. 4:**
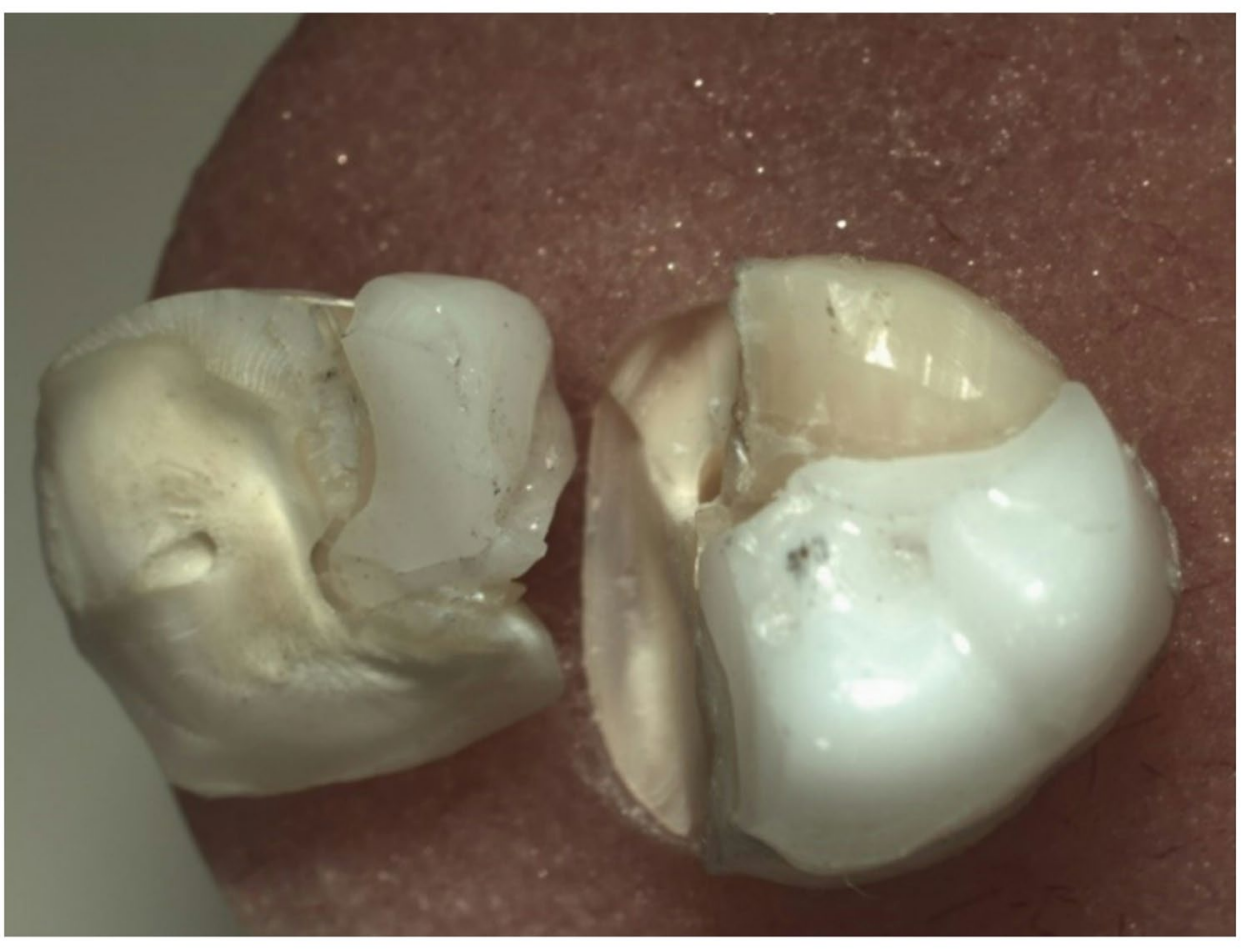
Type III failure pattern; Fracture of restoration and underlying tooth involving the pulp

Fractured specimens were analyzed using an optical microscope (SZ61TR, Model SZ2-ILST, Japan) based on fractographic principles to classify failure pattern [18, 31, 32] as follow; Type I: Fracture of restoration only. Type II: Fracture of the restoration and underlying tooth. Type III: Fracture of the restoration and underlying tooth involving the pulp (Table 3) and (Figs. 2, 3 and 4).

**Table 3:** Descriptive statistics for fracture resistance in (N) with failure pattern of all test groups

		Failure pattern		
Test groups	Mean ± SD (Newton)	Fracture of restoration (Type I)	Fracture of restoration with tooth (Type II)	Fracture of restoration with tooth involving pulp ) Type III)
Group ADC	4371 ± 440	5	5	0
Group FDC	3954 ± 698.3	3	3	0
Group ACS	3995 ± 274	3	3	1
Group FCS	3483 ± 567.3	4	4	0

**Fig. 5:**
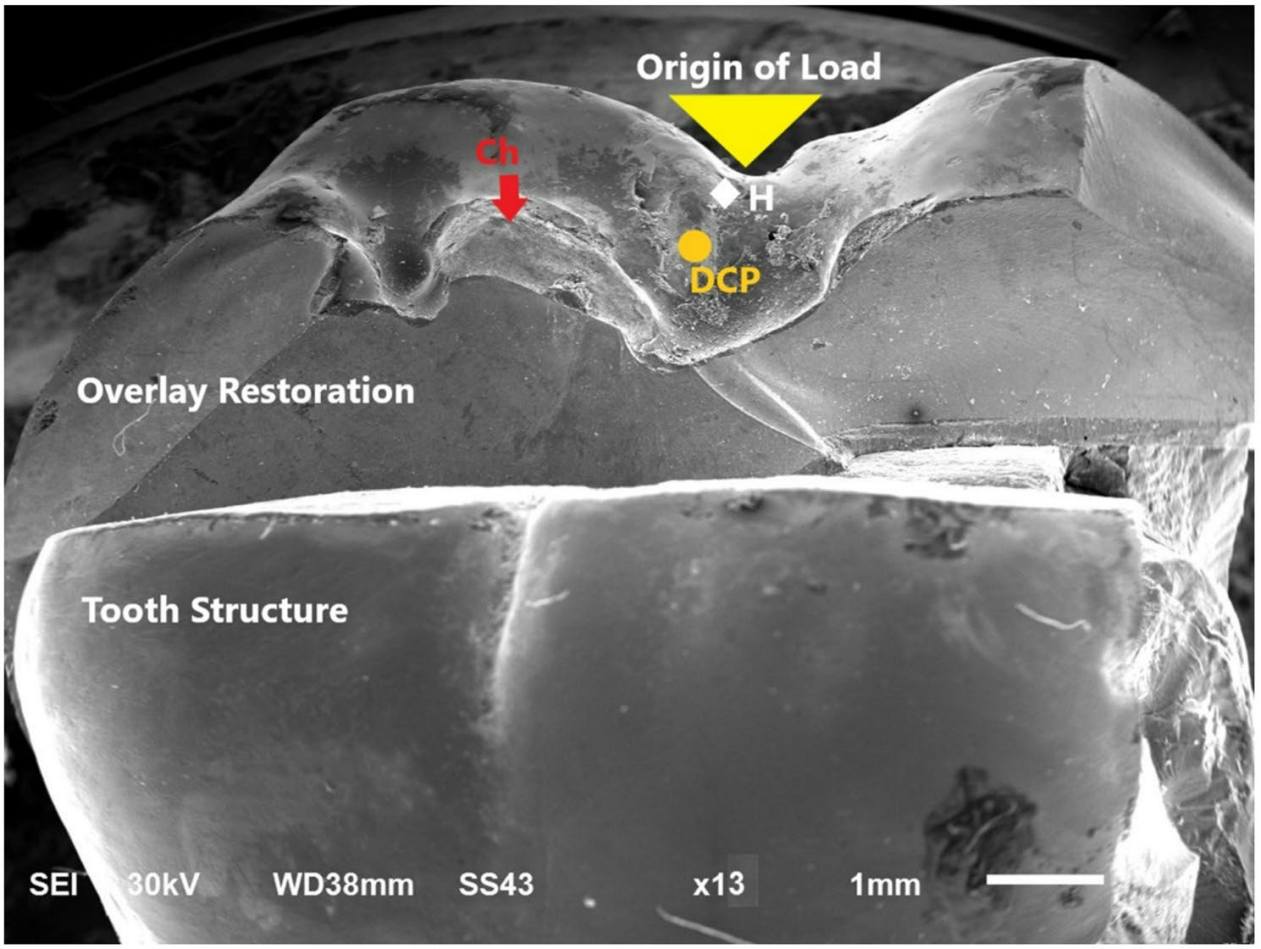
Representative SEM micrograph showing; Type I failure pattern. Magnification x13 Catastrophic fracture with chipping of the overlay restoration H = Hackle (origin of load) DCP = Direction of crack propagation CH = Chipping of overlay restoration

**Fig. 6:**
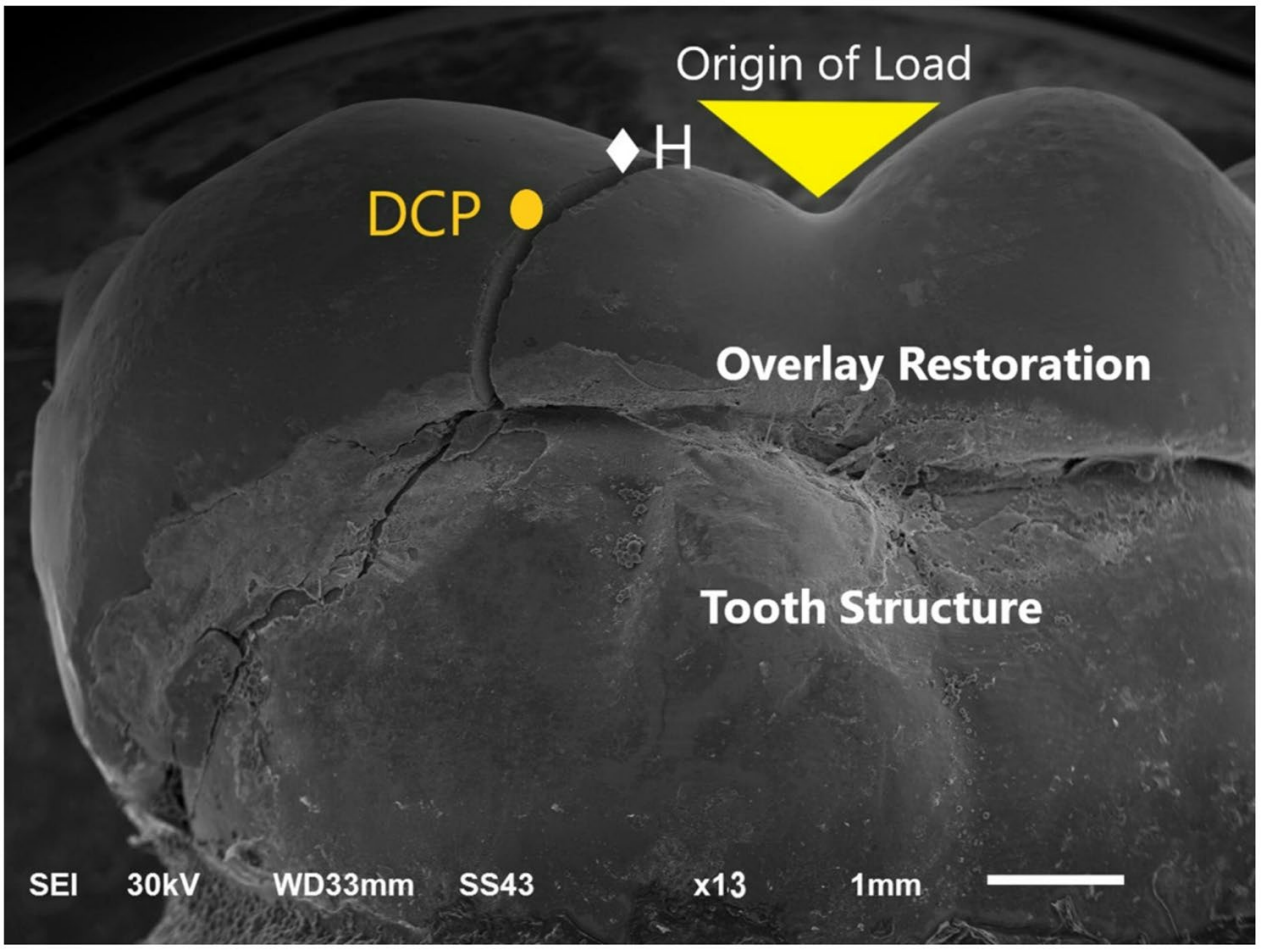
Representative SEM micrograph showing; Type II failure pattern. Magnification x13 Fracture with crack line involving overly restoration and underlying tooth H = Hackle (origin of load) DCP = Direction of crack propagation

**Fig. 7:**
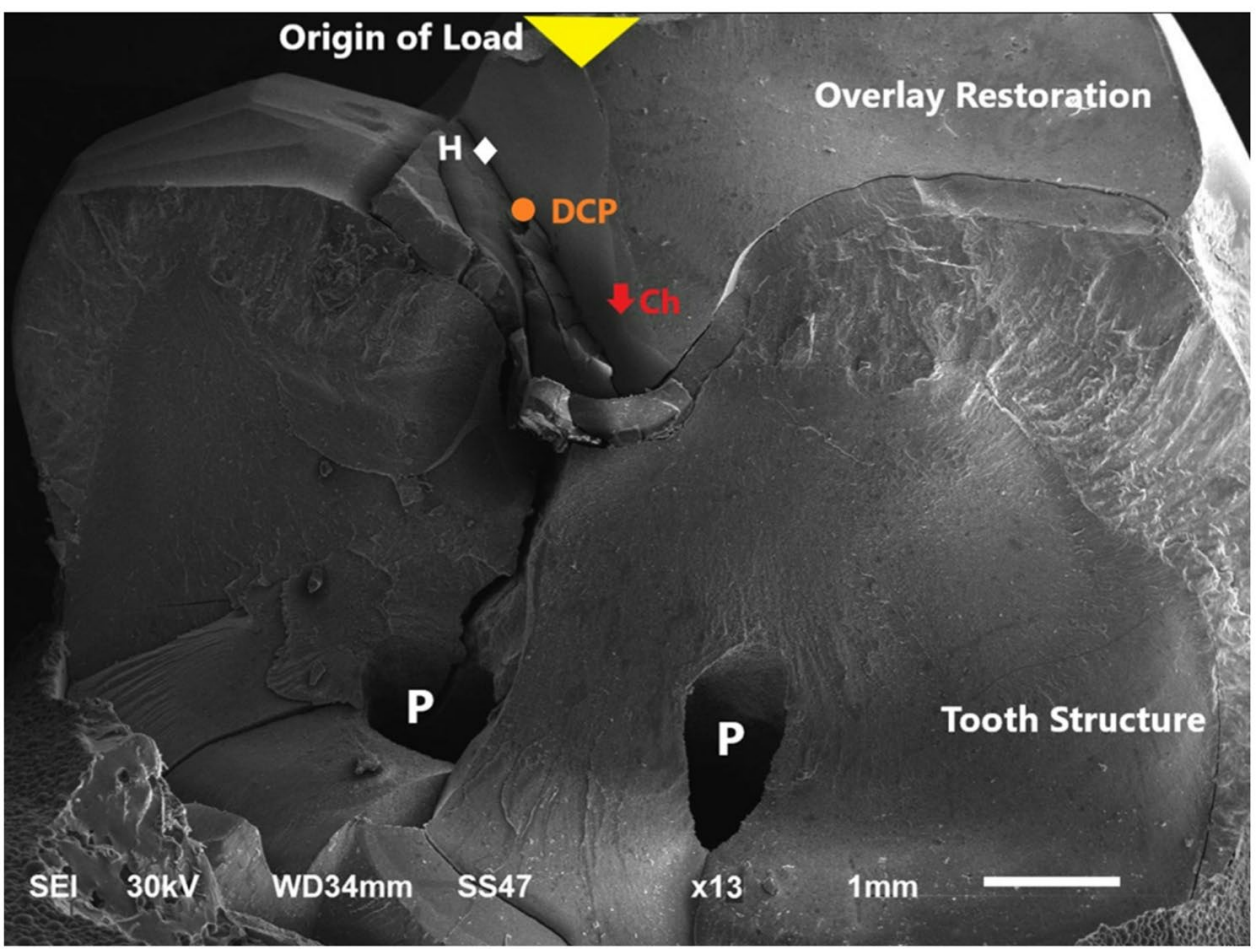
Representative SEM micrograph showing; Type III failure pattern. Magnification x13 Fracture with crack line involving overly restoration and underlying tooth involving the pulp H = Hackle (origin of load) DCP = Direction of crack propagation CH = Chipping of overlay restoration P = Pulp

A scanning electron microscope (SEM) (JEOL JSM 6510, JEOL Ltd, Japan) at magnifications X13 was utilized for further assessment of representative specimens for each failure pattern (Figs. 5, 6 and 7).

### Statistical analysis

Statistical analyses of the collected data were carried out using the Social Package for Statistical Science (SPSS) program, version 27.0. Two-way ANOVA and serial one-way ANOVAs at each study level were used for statistical analyses, followed by the Post Hoc Tukey test at *P* ≤ 0.05.

## Results

Two-way ANOVA model showed that the interaction of cusp design and cervical margin design was not significant (*P* = 0.8). While cusp design (*P* = 0.02) and cervical margin design (*P* = 0.04) were significant. Serial 1- way ANOVAs confirmed the results of 2-way ANOVA. Both cervical margin design (*P* = 0.04) and cusp design (*P* = 0.02) were statistically significant.

Fracture resistance (N) of group ACC showed the highest mean value of fracture load (4371 ± 440 N), followed by group ACS (3995 ± 274 N) and group FCC (3954 ± 567.3 N). The lowest fracture load was recorded for group FCS (3483 ± 567.3 N) (Table 3). For pairwise comparison between different studied groups, the Post Hoc Tukey test was performed at *P* ≤ 0.05. There were no statistically significant differences between the following test groups: (ACC, FCC, *P* = 0.39), (ACC, ACS, *P* = 0.48), (FCC, ACS, *P* = 0.99), (FCC, FCS, *P* = 0.29), (ACS, FCS, *P* = 0.22). There was statistically significant difference between test groups (ACC, FCS, *P* = 0.01).

### Analysis of failure patterns

No adhesive failure was observed, the main failure was catastrophic fracture among all groups (Type I), (Type II). One specimen in test group ACS showed fracture of restoration and underlying tooth involving the pulp (Type III).

## Discussion

According to the results of this in vitro study, the hypothesis was accepted because different preparation design of both cusps and cervical margin affected fracture resistance of overlay translucent zirconia restoration. However, all test group recorded fracture resistance values exceeding 3,483 N, which surpass the maximum masticatory forces typically exerted on posterior teeth clinically [20, 21].

Preparation for indirect overlay restorations remove only 32% to 47% from tooth structure [33]. This minimally invasive approach, plays a vital role in long term clinical durability of resin bonded overlay restoration [33]. Natural molars with similar dimensions were chosen to offer a more realistic replication of the clinical conditions [34]. Overlay restorations were CAD-CAM fabricated from 3Y-TZP translucent zirconia to ensure high mechanical properties of zirconia and homogeneous restoration thickness fabricated with CAD-CAM [35]. Bonding procedures were carried out in accordance with APC zirconia-bonding concept, to ensure longevity of resin bonded overlay zirconia restorations [36]. To simulate clinical conditions, the restorations were stored in water for 45 days followed by cyclic loading fatigue for 120000 cycles to mimic one year clinically.

Comparing the fracture resistance values in this study to those reported in previous research is challenging. Cusp design plays a critical role in the ability of restored teeth to withstand masticatory forces [37]. Group ACC (anatomical cusp with chamfer finish line) exhibited the highest fracture resistance among all test groups (4317 N). Anatomical cusp design, is a conservative approach that closely mimics the natural contour of the tooth, consequently preserve remaining tooth substrate [33]. Also, the orientation of cusp reduction plays a crucial role in the biomechanical performance of restorations [37]. Reduction according to natural inclines of the cusps, provide uniform distribution of masticatory forces on the bonded restoration tooth assembly [38]. Consequently, stress concentrations were reduced, and the risk of fracture was minimized [37, 38]. On the other hand, in case of flat cusps reduction, reducing cusp inclinations might affect fracture resistance, as it alters the natural anatomy that helps to direct occlusal forces along the long axis of posterior teeth, increasing the risk of crack and fracture of resin bonded ceramic and the underlying tooth substrates [38]. Several published literatures reported that different cusp preparation designs affected fracture resistance of overaly, vonlay and onlays resin bonded restorations fabricated from different types of ceramic materials [18, 30, 32]. Other studies [12, 39, 40] concluded that anatomical reduction of occlusal surface exhibited the highest fracture resistance values. Abdelaal et al., [41] found that overlay design demonstrated a higher fracture resistance compared to vonlay design although they used epoxy resin dies compared to natural molars in this in vitro study. However, these results are contrary to the results of Attia et al., [42] who concluded that preparation design had no effect on fracture resistance. This difference could be attributed to the fact that they used lithium disilicate and hybrid ceramics, in comparison to monolithic translucent zirconia used in this study.

Moreover, results of this in-vitro study proved that increasing thickness of the overlay restoration positively influenced fracture resistance. These results are contradicting to results of Chen et al., [43] who report that a thickness of 1 mm for overlay restoration was more effective than 1.5 mm. This difference could be attributed to many factors such as they used different tooth preparation designs, as well as different ceramic materials such as lithium disilicate glass ceramics and machinable composite resin. Additionally, different artificial aging regimes used in both studies could be another factor.

Different margin designs affected fracture resistance of teeth restored with resin bonded overlay restorations [18, 31]. Chamfer margins are easily identifiable and provide favorable visibility during preparation [39]. Moreover, they enhance resistance to masticatory forces and minimize the risk of chipping of restoration at cervical margin [39]. A chamfer finish line helps to distribute stress more evenly across the restoration and the tooth structure [44]. This design creates a rounded, convex internal angle that prevents the concentration of forces and improves the overall durability of the restoration [45]. Additionally, a properly prepared chamfer provide adequate bulk of the restoration at cervical margins to withstand masticatory forces [45, 46]. In contrast, a gingival step preparation, characterized by complex geometries with sharp internal angles, particularly at the axiopulpal junction, these sharp angles led to stress concentrations, that might increased the risk of fracture under repeated masticatory forces [12, 47]. Therefore, the preparation design of group ACC (anatomical cusp with chamfer finish line) demonstrated the highest fracture resistance (4317 N), while group FCS (Flat cusp with gingival step) recorded the lowest value (3483 N). Interestingly, the lowest fracture resistance value recorded for FCS group, was still higher than the normal masticatory forces [48]. The recorded high fracture resistance of all test groups in this study could be attributed to the supreme mechanical properties of monolithic translucent zirconia used for fabricating overlay restorations as reported in other study [1]. Another factor could be bonding the restorations were strictly following APC bonding protocol which ensured durable adhesive bonding of restoration/tooth assembly [36] making them reacting against the applied fracture load as one unit [20, 21, 23].

Considering the effect of artificial ageing, prolonged exposure to water can induce hydrolytic degradation, which may compromise the integrity of the bond between the restoration and the tooth substrate. Furthermore, cyclic loading in wet conditions, can lead to the development of microcracks within the ceramic and accelerate propagation of inherent microcracks [20, 21]. These microcracks are critical as they could contribute to catastrophic fractures that compromise the bonded restoration [1]. Moreover, cyclic loading fatigue might cause deboning of the restoration, because ceramic/luting cement/tooth interferes are considered the weakest point in indirect bonded restorations [20, 21].

Considering failure pattern, fractured specimens of all test groups exhibited type I and type II fracture patterns except one specimen from group (ACS). These failure patterns involved cracks or fractures of the restoration and tooth substrates without pulp involvement. Both type I and II failure patterns were considered favorable because they did not compromise the fundamental health of the restored teeth. However, one specimen from test group (ACS) including anatomical cusp reduction and gingival steps exhibited type III failure pattern with pulp involvement. This specific type of failure was considered unfavorable because it extended below CEJ and might necessitate tooth extraction as reported in other studies [1, 18, 20, 31, 32].

This study has several limitations, primarily due to its in vitro design. To obtain more accurate results, clinical studies are recommended. Furthermore, this research investigated only single type of zirconia and one resin cement. Future studies should investigate a wider variety of ceramic materials and luting cements. Additional research is needed to evaluate fracture resistance after long term water storage, and thermalcycling under repeated cyclic loading fatigue instead of compressive load.

## Conclusions

Within the limitations of this in vitro study, the following findings can be concluded:

1. Both cusp design and margin design influenced fracture resistance of molars restored with monolithic translucent zirconia overlay restorations.
2. Anatomical cusp design with a chamfer finish line showed the highest fracture resistance.
3. Flat cusp design with a gingival step showed the lowest fracture resistance.

## Data Availability

The data sets used and/or analyzed during the current study are available from the corresponding author upon reasonable request.

## Abbreviations

PIAR: Posterior indirect adhesive restorations
N: Newton
SEM: Scanning electron microscope
MOD: Mesial-occlusal distal
CEJ: Cemento-enamel junction
SPSS: Social Package for Statistical Science
CAD/CAM: Computer aided design; computer aided manufacture
